# Exosomes from Von Hippel-Lindau-Null Cancer Cells Promote Metastasis in Renal Cell Carcinoma

**DOI:** 10.3390/ijms242417307

**Published:** 2023-12-09

**Authors:** Kailey Flora, Moe Ishihara, Zhicheng Zhang, Elizabeth S. Bowen, Aimee Wu, Tala Ayoub, Julian Huang, Celine Cano-Ruiz, Maia Jackson, Kaveeya Reghu, Yasmeen Ayoub, Yazhen Zhu, Hsian-Rong Tseng, Z. Hong Zhou, Junhui Hu, Lily Wu

**Affiliations:** 1 Department of Bioengineering, University of California, Los Angeles, CA 90095, USA; kaileyflora@gmail.com; 2 Department of Molecular and Medical Pharmacology, David Geffen School of Medicine, University of California, Los Angeles, CA 90095, USA; mishihara@mednet.ucla.edu (M.I.); zzc168439zzc@gmail.com (Z.Z.); celinecanoruiz@g.ucla.edu(C.C.-R.); 3 Department of Computational and Systems Biology, University of California, Los Angeles, CA 90095, USA; ebowen19@g.ucla.edu; 4 Department of Molecular, Cell and Developmental Biology, University of California, Los Angeles, CA 90095, USA; aimeeywu@g.ucla.edu (A.W.); julianhuang@g.ucla.edu (J.H.); maiajackson@g.ucla.edu (M.J.); kreghu42@g.ucla.edu (K.R.); 5 Department of Physiology, University of California, Los Angeles, CA 90095, USA; tala.ayoub@g.ucla.edu; 6 School of Medicine, Saint Louis University, St. Louis, MO 63104, USA; yasmeen.ayoub@health.slu.edu; 7 California NanoSystems Institute, Crump Institute for Molecular Imaging, Department of Molecular and Medical Pharmacology, University of California, Los Angeles, CA 90095, USA; yazhenzhu@mednet.ucla.edu (Y.Z.); hrtseng@mednet.ucla.edu (H.-R.T.); hong.zhou@ucla.edu (Z.H.Z.); 8 Department of Pathology and Laboratory Medicine, David Geffen School of Medicine, University of California, Los Angeles, CA 90095, USA; 9 Jonsson Comprehensive Cancer Center, David Geffen School of Medicine, University of California, Los Angeles, CA 90095, USA; 10 Department of Microbiology, Immunology and Molecular Genetics, University of California, Los Angeles, CA 90095, USA; 11 Department of Urology, David Geffen School of Medicine, University of California, Los Angeles, CA 90095, USA

**Keywords:** exosomes, metastasis, renal cell carcinoma, EMT, cell–cell communication, CAM model

## Abstract

Exosomes are extracellular vesicles that modulate essential physiological and pathological signals. Communication between cancer cells that express the *von Hippel-Lindau (VHL)* tumor suppressor gene and those that do not is instrumental to distant metastasis in renal cell carcinoma (RCC). In a novel metastasis model, VHL(−) cancer cells are the metastatic driver, while VHL(+) cells receive metastatic signals from VHL(−) cells and undergo aggressive transformation. This study investigates whether exosomes could be mediating metastatic crosstalk. Exosomes isolated from paired VHL(+) and VHL(−) cancer cell lines were assessed for physical, biochemical, and biological characteristics. Compared to the VHL(+) cells, VHL(−) cells produce significantly more exosomes that augment epithelial-to-mesenchymal transition (EMT) and migration of VHL(+) cells. Using a Cre-*loxP* exosome reporter system, the fluorescent color conversion and migration were correlated with dose-dependent delivery of VHL(−) exosomes. VHL(−) exosomes even induced a complete cascade of distant metastasis when added to VHL(+) tumor xenografts in a duck chorioallantoic membrane (dCAM) model, while *VHL*(+) exosomes did not. Therefore, this study supports that exosomes from VHL(−) cells could mediate critical cell-to-cell crosstalk to promote metastasis in RCC.

## 1. Introduction

Kidney cancer is the seventh most common type of cancer in the United States [1,2]. Projections estimate 81,800 new cases and 14,890 deaths related to kidney and renal pelvis cancer in 2023. The most common subtype of kidney cancer is clear cell renal cell carcinoma (ccRCC), accounting for roughly 68% of all kidney cancer cases [3,4]. About 40% of all RCC patients are at risk of developing metastasis. One-third of them present with metastatic disease, and 30% of patients with localized tumors could develop distant metastasis later in the course after surgery. Once metastasized, the 5-year survival rate drops precipitously to approximately 12% [5,6]. Therefore, there is a clear, urgent need to develop more effective diagnostic and therapeutic approaches for metastatic ccRCC through a better understanding of the molecular mechanism of metastasis.

Loss of function of the *von Hippel-Lindau (VHL)* tumor suppressor gene, observed in 70% of ccRCC cases, is considered an early oncogenic driving event in this malignancy. The VHL protein, pVHL, plays an essential role in the mammalian oxygen-sensing pathway and regulates the expression of hypoxia-inducible factors (HIF1α and HIF2α) via ubiquitin-mediated degradation [7,8,9]. In the absence of pVHL, HIF1α and HIF2α are stabilized and induce the hypoxia response program, such as angiogenesis [10,11]. Consequently, ccRCC tumors are highly vascularized, with high metastatic potential frequently disseminating to the lungs [12,13,14]. Despite that the *VHL* gene mutation is considered to be an early oncogenic event in ccRCC, our recent work shows that the expression of pVHL in human ccRCC is heterogeneous, whereby population with the presence (VHL(+) cells) and the absence of wildtype pVHL (VHL(−) cells) are observed to coexist side by side in the same tumor [15]. This heterogeneity of VHL loss within one tumor may be due to the simultaneous development of two cell populations, each developed from a precursor cell where VHL loss did or did not occur. Preclinical models of ccRCC showed that distant lung metastasis required co-existence of and cooperation between VHL(+) and VHL(−) cells [15,16]. Specifically, VHL(−) cells serve as metastasis drivers, as they increase the aggression and growth of VHL(+) cells. This work suggests that intercellular communication between these two distinct cell populations could transmit critical metastatic signals.

Exosomes are a type of extracellular vesicle secreted by a wide range of cell types that can act locally or disperse via circulation and act at distant sites [17,18,19]. Exosomes can carry distinct cargos containing cell- and environment-specific bioactive materials to transmit instructive signals between cells [17], altering gene expression and cellular pathways and functions in recipient cells. A large amount of evidence showed that exosomes produced by different cells in a tumor microenvironment, such as stroma, macrophages, and cancer cells themselves, can facilitate metastasis [20,21,22,23]. One of the explanations is that, in hypoxic environments like tumors, cells produce increased exosomes compared to cells in a normoxic environment [24]. Hypoxia-derived exosomes regulate the expression of genes associated with angiogenesis, epithelial-to-mesenchymal transition (EMT), and metastasis, adapting the cells to the hypoxic environment by promoting their motility, escape, and survival of the unfavorable conditions [24,25]. Due to the absence of a functioning pVHL to downregulate the hypoxic response, VHL(−) cells are in a pseudo-hypoxic state and produce more exosomes than their counterparts [25]. As a result, exosomes originating from VHL(−) cells may act as a driver of metastasis in heterogeneous ccRCC tumors. Therefore, this study assessed the functional capabilities of exosomes from VHL(+) and VHL(−) cells to understand their contribution to the pro-metastatic cross-communication in heterogeneous ccRCC tumors.

## 2. Results

### 2.1. Physical and Biochemical Characterization of RCC Exosomes

VHL(+) and VHL(−) pairs were created from two RCC cell lines: one murine, Renca, and the other human, ACHN. Both Renca and ACHN have a wildtype *VHL* gene and express functional pVHL. So, we utilized lenti-CRISPR to stably knock out the *VHL* gene [15,16]. Hereafter, the parental cell lines with wildtype pVHL are denoted as RC-VHL(+) and AC-VHL(+), respectively, while the *VHL* knockout lines are denoted as RC-VHL(−) and AC-VHL(−), respectively.

**Figure 1:**
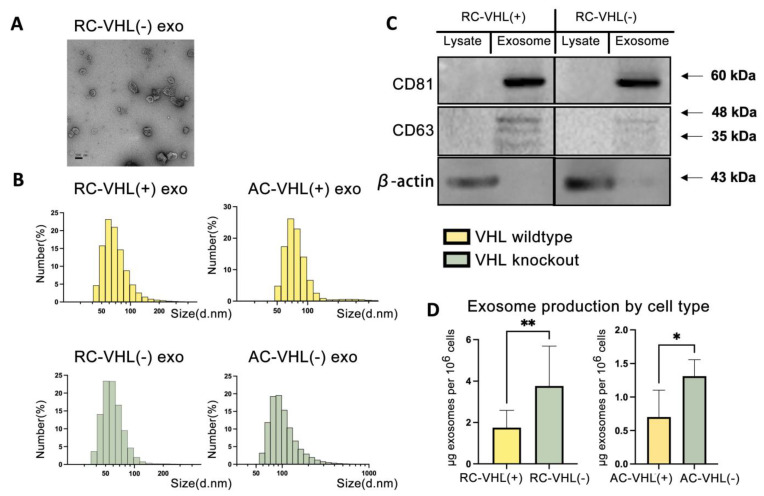
Physical and biochemical characterization of RCC exosomes. (**A**) TEM imaging of RC-VHL(−) exosomes. Scale bars: 100 nm. (**B**) DLS of tumor-derived exosomes. (**C**) Western blotting of lysates and exosomes from RC-VHL(+) and RC-VHL(−) for exosome-specific markers CD81 and CD63. β-actin was used as the loading control for cellular lysates. (**D**) Exosome production in RCC cell types measured by bicinchoninic acid (BCA) assay. Statistical analysis using Welch’s *t*-test (* *p* < 0.05, ** *p* < 0.01).

We first analyzed exosomes’ physical and biochemical properties derived from these RCC cell lines. Transmission electron microscopy (TEM) showed the morphology of RCC exosomes in concordance with typical exosome morphology, which has a dent in the center and is sized between 30 and 100 nm [26] (Figure 1A, Supplementary Figure S1D). Subsequently, we performed dynamic light scattering (DLS) to determine the size distribution profile of the RCC exosomes (Figure 1B). DLS number measurements were used instead of intensity measurements to avoid artifactual results due to exosome aggregation [27]. The highest proportion of RC-VHL(+) and AC-VHL(+) were 58.8 nm and 68.0 nm, respectively. Within VHL(−) exosomes, the highest proportion of RC-VHL(−) was 50.7 nm, and AC-VHL(−) exosomes peaked at 78.8 and 91.3 nm. All of these DLS results showed a Gaussian exosome size distribution from 50 to 150, falling within the expected size range of exosomes [28].

To assess the constituents of the harvested exosomes, we performed Western blotting for classical exosome markers, CD63 and CD81, which are common membrane-spanning tetraspanins enriched in exosome membranes (Figure 1C) [29]. Strong bands for CD63 and CD81 were seen in exosome samples compared to cell lysates from corresponding Renca cell lines. CD63 produces multiple bands due to the multiple glycosylated isoforms from post-translational modifications [30]. Similarly, CD63 and CD81 were also seen in the exosome preparation of ACHN cells (Supplementary Figure S1A). The control β-actin is a ubiquitous cytoskeletal component that is typically absent in exosomes. Strong bands were seen solely in cell lysate samples and not in exosome samples, suggesting our protocol achieved effective exosome isolation from cellular components for both the Renca and ACHN lines.

The exosome concentration significantly differed between VHL-null cells and cells with wildtype pVHL (Figure 1D). In Renca, RC-VHL(+) produced, on average, 1.75 µg exosomes per 10^6^ cells, while RC-VHL(−) produced 3.77 µg per 10^6^ cells, resulting in more than two-fold differences (*p*-value = 0.0082). Similarly, AC-VHL(−) produced approximately twice the number of exosomes, with 1.12 µg exosomes per 10^6^ cells compared to 0.62 µg of AC-VHL(+) counterparts (*p*-value = 0.0019). Since VHL-null cells are in a pseudo-hypoxic state due to upregulation of the HIF1α and HIF2α, these concentration differences support previous studies claiming that cells in a more hypoxic environment generate more extracellular vesicles [15].

### 2.2. VHL(−) Exosomes Promote VHL(+) Migration and Invasion

**Figure 2:**
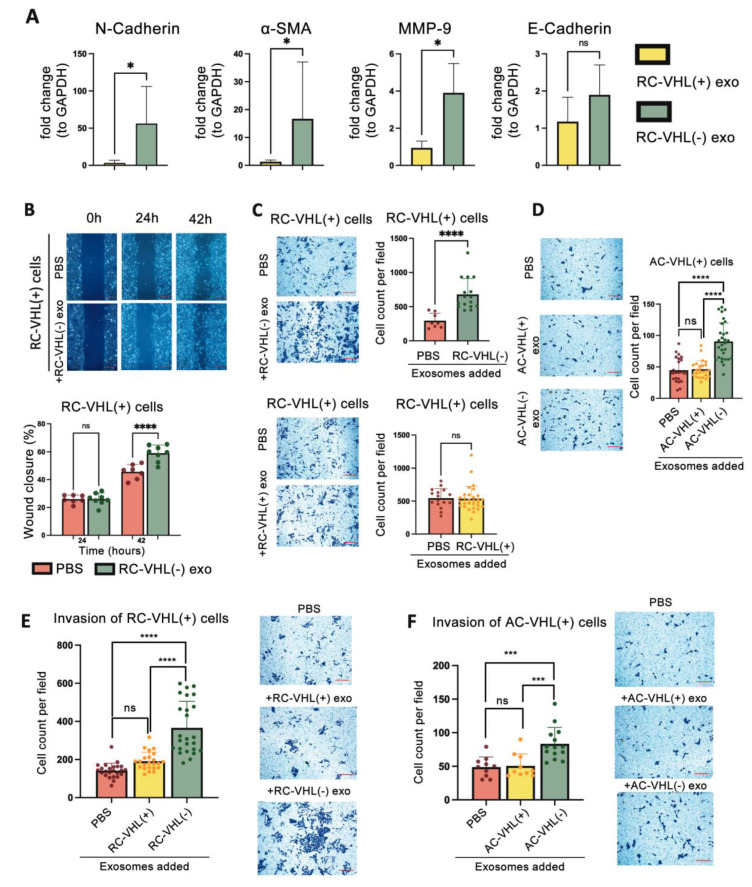
VHL(−) exosomes promote VHL(+) EMT, migration, and invasion. (**A**) EMT marker RNA in VHL(+) cells upon 50 h incubation with VHL(+) or VHL(−) exosomes. (**B**) Wound healing assay of RC-VHL(+) cells treated with PBS or RC-VHL(−) exosomes for 42 h. (**C**–**F**) Transwell 3D migration and invasion assays of VHL-WT cells in response to VHL-KO and VHL-WT exosomes after 36 h incubation (**C**,**E**) and AC- VHL(+) cells in response to AC-VHL(+) and AC-VHL(−) exosomes after 50 h incubation (**D**,**F**). All images were taken at 10× magnification using a light microscope. Scale bars: (**A**) 100 μm, (**B**–**F**) 500 μm = 100 u. Statistical analysis was completed using (**A**) unpaired *t*-test, (**C**) Welch’s *t*-test, and (**B**,**D**–**F**) ordinary one-way ANOVA (* *p* < 0.05, *** *p* < 0.001, **** *p* < 0.0001, ns: not significant).

Upon epithelia-to-mesenchymal transition (EMT), cells typically attain mesenchymal markers, lose epithelial markers, and exhibit more migratory and invasive behaviors [31,32]. We enquired whether EMT could be a sequela of exosome-mediated cross-communication. RC-VHL(+) cells were incubated with exosomes harvested from either RC-VHL(+) or RC-VHL(−) cells. Incubation with RC-VHL(−) exosomes resulted in a significant increase in mesenchymal marker proteins, such as N-cadherin, α-SMA, and MMP-9, in comparison to RC-VHL(+) exosomes (Figure 2A). Of interest, EMT transcription factors such as SNAI1 (Snail1), SNAI2 (Slug), ZEB1, and ZEB2, and epithelial marker E-cadherin (Figure 2A) were not significantly altered by exosome treatment.

**Figure 3:**
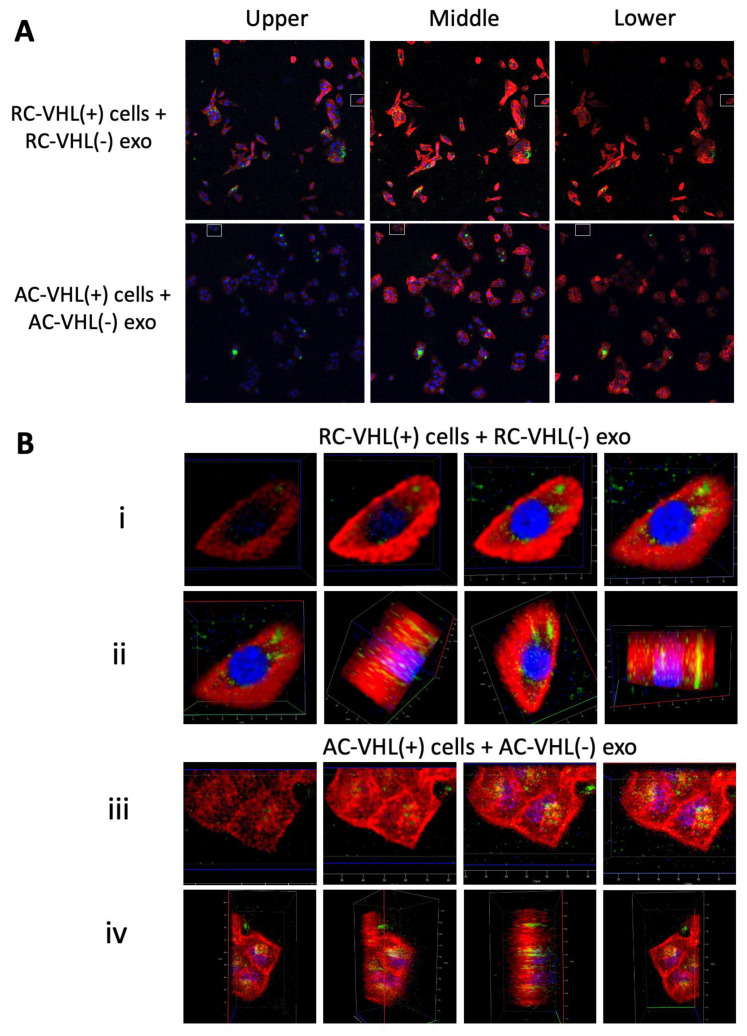
VHL(−) exosomes are internalized into VHL(+) cells. Three-dimensional confocal imaging of (**A**) RC-VHL(+) cells with RC-VHL(−) exosomes and AC-VHL(+) cells with AC-VHL(−) exosomes shown as layers moving through z plane from upper to lower view. White boxes are used for closeups of small cell clusters in (**B**)**,** in i and iii**,** layers of the z plane, and ii and iv**,** rotation about the *x*-axis. Confocal imaging was completed at 10× magnification.

**Figure 4:**
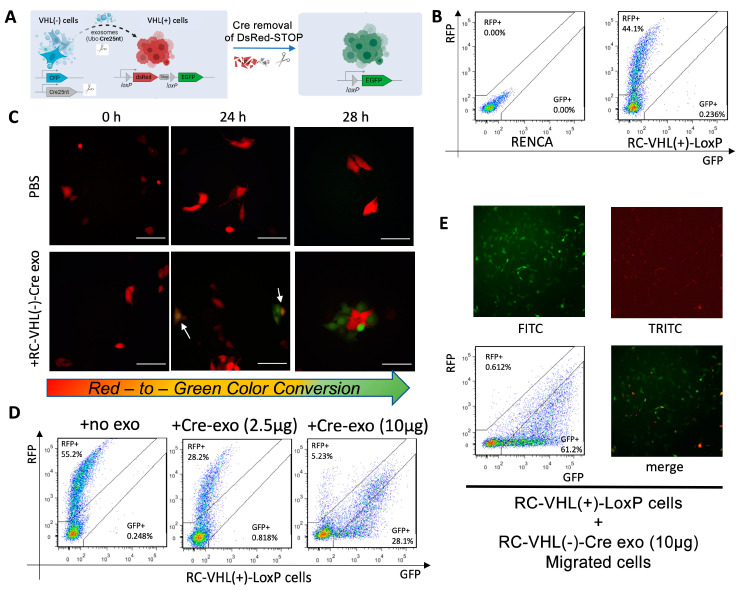
The Cre-*loxP* system enables exosome-mediated color conversion. (**A**) Schema of the Cre-*loxP* system. (**B**) Flow cytometry for RFP and GFP signals in the parental RENCA line and VHL-WT-*loxP*. (**C**) Color conversion of VHL-WT-*loxP* cells from red to green fluorescence upon incubation with VHL-KO-Cre exosomes over a 28 h course. Arrows indicate cells in the process of color conversion. Fluorescent microscope, 20× magnification, scale bars: 100 μm. (**D**) Quantification of fluorescent VHL-WT-*loxP* cells using flow cytometry upon treatment with various amounts of VHL-KO-Cre exosomes at 48 h. (**E**) Microscope images and flow cytometry quantification of the VHL-WT-*loxP* cells migrated through the transwell to the bottom chamber, which are predominantly GFP^+^. Fluorescent microscope, 4× magnification.

Next, we assessed whether exosomes could increase the migration of VHL(+) cells in two-dimensional and three-dimensional assays. Over the course of 42 h, RC-VHL(+) cells with RC-VHL(−) exosome treatment had increased two-dimensional migration compared to the control group of RC-VHL(+) cells treated with PBS (Figure 2B). At 24 h, there was no significant difference between the control and experimental groups, likely due to the initial time needed for exosome to internalize and act, estimated to be about 24 h (see Figure 3 and Figure 4 below). The VHL(−) exosomes exerted a discernible increase in the migration of RC-VHL(+) cells in closing the gap over the last 18 h of the incubation. The three-dimensional migration capability of VHL(+) cells was tested by comparison of migration across pores in transwell inserts by chemotaxis towards exosome-depleted FBS in the absence and the presence of added VHL(−) and VHL(+) exosomes (Figure 2C). In the presence of RC-VHL(−) exosomes, RC-VHL(+) cells have increased migratory abilities, while RC-VHL(+) exosomes had no significant effect. Furthermore, these results were corroborated in the ACHN human cell line over a 50 h incubation period, and the AC-VHL(−) exosome-treated groups had approximately twice the number of migratory cells (Figure 2D). Invasive behavior of exosome-treated cells to migrate through the extracellular matrix layer in transwell inserts was tested. In both Renca and ACHN cells, VHL(+) exosomes exerted no significant effect, while VHL(−) exosomes caused approximately a 1.5–2.0× increase in the number of cells invading across the matrix (Figure 2E,F).

Next, we assessed whether exosomes could increase cellular proliferation, which could, in turn, contribute to the increased migration and invasion observed. The proliferation of RC-VHL(+) and AC-VHL(+) cells, measured by MTS tetrazolium assay, remained unchanged after 45 h incubation of VHL(+) exosomes or VHL(−) exosomes (Supplementary Figure S2). These results suggest that RCC exosomes do not increase cellular proliferation, but exosomes from VHL(−) induce an EMT-like phenotype in VHL(+) cells, with upregulation of some of the EMT markers and increased migration and invasion. However, these effects are non-reciprocal, as VHL(+) exosomes did not induce any significant changes in the VHL(−) cells’ migratory or invasive behavior (Supplementary Figure S3). This collaborates with our previous findings that VHL(−) cells are the metastatic driver that transforms the recipient VHL(+) cells into a more aggressive state and contributes to distant metastasis [15].

### 2.3. Exosome Uptake

Next, we assessed the physical uptake of VHL(−) exosomes into the VHL(+) cells that initiate the pro-metastatic cross-communication. To ascertain the uptake process, VHL(−) exosomes were stained with PKH67. After a 24 h incubation with the exosomes, two-dimensional images show exosomes within the boundary of the cell, as the green fluorescence (PKH67) is localized within the cytoplasm instead of at the cell membrane where red fluorescence staining of fibrous actin in the cytoskeleton is prevalent (Supplementary Figure S4). To confirm that exosomes are internalized and localized near the nucleus (DAPI, blue), three-dimensional imaging was performed (Figure 3). Separation of *z*-axis layers into upper, middle, and lower x-y images show that the highest presence of green exosomes can be seen near the blue, DAPI-stained nucleus, as when the red, F-actin-stained cytoskeleton is in primary view in the lower layer, there is much less green fluorescence than in the upper and middle layers (Figure 3A). By selecting smaller sections of the original images, a clearer view of exosome internalization can be observed (Figure 3B). Within the upper, middle, and lower x-y images through the z-dimension, one can see that peak green exosome fluorescence appears with peak blue nucleus fluorescence. Additionally, when rotated about the *x*-axis to view the z-dimension, this further corroborates that the exosome signal appears at the same dimension as the nucleus and within the boundaries of the red cytoskeleton. These images suggest that 24 h is sufficient for the internalization of exosomes into the cell for both the Renca and ACHN cell lines. Additional three-dimensional imaging of VHL(+) cells with PKH67-stained VHL(−) exosomes shows that the exosomes localize within the cell, often surrounding the DAPI-stained nucleus (Supplementary Video S1–S6).

### 2.4. Visualization of Exosome Internalization and Induced Migration Using a Cre-loxP System

To assess if internalized exosomes have a functional migration impact on the recipient cells, a Cre-*loxP* exosome reporter system was devised [33,34] (Figure 4A). The Cre-*loxP* system has Cre recombinase inserted into RC-VHL(−) cells, resulting in RC-VHL(−)-Cre cells, while the VHL(+)-*loxP* cells contained genes with red fluorescent protein followed by a stop signal and then green fluorescent protein gene (RFP-STOP-GFP). In this system, the color of RC-VHL(+)-*loxP* cells changes from red to green when VHL(−)-Cre exosomes are internalized, and the released Cre could then remove the RFP via recombination (Figure 4B). The physical, biochemical, and cellular properties of the RC Cre-*loxP* reporter system were comparable to the parental RC-VHL(−)and RC-VHL(+) cells (Supplementary Figures S1B, S2B, S3B,D,F, S4B and S5).

Exosomes from RC-VHL(−)-Cre cells were added to the RC-VHL(+)-*loxP* cells at time 0, at which time, the recipient cells maintained expression of RFP (Figure 4C); after 24 h, some of the recipient cells had completed the color change and expressed GFP, while other cells still likely expressed both RFP and GFP and appeared yellow–orange. This result suggests a 24 h window is needed for exosomes to internalize and become functional in recipient cells. At hour 28, the number of cells expressing GFP increased, as more RC-VHL(+)-*loxP* cells had completed the Cre-induced color conversion. Flow cytometry confirmed these findings, showing virtually no GFP conversion without RC-VHL(−)-Cre exosome addition, then an increase in GFP concurring with a decrease in RFP upon exosome addition in a dose-dependent manner (Figure 4D). This exosome uptake was shown to be the direct cause of the increased migration of RC-VHL(+)-*loxP* cells when we isolated the cells in the bottom chamber of the transwell assay (Supplementary Figure S5D). The bottom chamber of the transwell assays contained the cells with upregulated migration, and flow cytometry showed that the migrated population was predominantly GFP positive or a transient yellow color rather than RFP (61.2% vs. 0.612%; Figure 4E). Therefore, the results of the Cre-*loxP* exosome uptake reporter system support the direct causal relationship between exosome uptake and the upregulation of migratory function in recipient cells.

### 2.5. VHL(−) Exosomes Promote RCC Metastasis in Ovo

**Figure 5:**
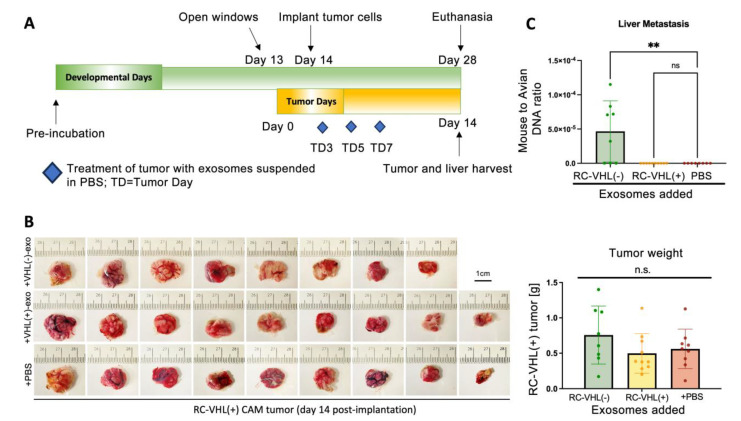
VHL(+)-exo induces distant metastases of VHL(+) tumors in an animal model. (**A**) Schema of the dCAM model. (**B**) Gross pictures of RC-VHL(+) tumors formed in dCAM and their weight analysis. (**C**) qPCR analysis of dCAM livers measuring mouse to avian β-actin ratio, indicative of distant metastases. Statistical analysis was completed using (**A**) ordinary one-way ANOVA and Dunnett’s test (** *p* < 0.01, ns: not significant).

Next, we assessed the impact of exosome treatment on in ovo tumor growth and metastasis in a duck chorioallantoic membrane model (dCAM). CAM models are less expensive and time-consuming than immunocompromised mice in assessing cancer metastasis [35,36,37]. The dCAM model has a short tumor growth window of two weeks after implantation, an adequate time for aggressive tumor cells to escape from the primary tumor and disseminate via circulation to distant organs and establish micro-metastases in the embryo (Figure 5A). RC-VHL(+) cells were treated with no exosomes, RC-VHL(+) exosomes, or RC-VHL(−) exosomes, given three times throughout the remaining developmental window, from developmental day 14 to day 28. On the day of euthanasia, after 14 days of tumor growth, the average size of the primary tumors and liver metastases were analyzed. The liver is the most vascularized organ of the embryo and has high potential as a metastatic site. This differs from clinical RCC in humans, where lung metastasis is the most common metastatic site [13,14]. This difference is because embryonic lungs have not inflated and are less vascularized than the liver prior to hatching; consequently, it is less likely to be a metastatic site [38].

The average weights of RC-VHL(+) CAM tumors after a 14-day incubation were 0.6 g, 0.5 g, and 0.75 g for the PBS, RC-VHL(+)-exo, and RC-VHL(−)-exo treatment groups, respectively, and did not reach a statistical significance (Figure 5B, Supplementary Figure S6). This result was consistent with the findings of in vitro proliferation assays (Supplementary Figure S2). The extent of murine RC cells metastasized to the liver was assessed by the ratio of murine to avian DNA using quantitative PCR (Figure 5C). Both the control RC-VHL(+) cell-only group and the RC-VHL(+)-exo treatment group showed no detectable level of mouse β-actin DNA. Only the RC-VHL(−)-exo-treated group showed a detectable level of mouse DNA in the duck embryo liver, significantly higher than the other two treatment groups. These results corroborate the in vitro data where exosomes from VHL(−) but not VHL(+) cells are able to induce more migratory and invasive behaviors of VHL(+) cells (Figure 3). Moreover, in vitro phenotypic changes directly contribute to metastatic function in vivo, leading to distant metastases and completing the full metastatic cascade in the animal model.

## 3. Discussion

Even though intratumoral gene expression heterogeneity is a feature widely observed in ccRCC [39], the functional significance of this heterogeneity is not fully defined. Our recent study showed that intratumoral heterogeneity extends to the *VHL* gene, which is identified as an early genetic mutation event in the oncogenesis of ccRCC [15]. We demonstrated that a functional significance of the VHL heterogeneity, namely the co-existence of cells expressing wildtype VHL protein (VHL(+)) and non-expressing ones, VHL(−) cells contribute directly to metastatic progression by a cooperative mechanism [15]. In this novel mechanism, both VHL(−) and VHL(+) cells are necessary to produce distant metastasis, where the VHL(−) cells drive metastasis by inducing the proliferation, EMT, and increased migratory function of VHL(+) cells via soluble factors such as periostin (18). This study is a follow-up of our prior study to ask whether exosomes produced by VHL(−) cells could also be driving metastatic progression in ccRCC. In summation, the answer to this inquiry is clearly positive. First, VHL(−) cells secrete significantly more exosomes than VHL(+) cells, which is likely attributed to the pseudo-hypoxic state in the VHL(−) cells secondary to the loss of the VHL gene [11,15,16,24,25]. Further, the functional capabilities of the exosomes produced by each cell type are distinctly different. Treating VHL(+) cells with VHL(−) exosomes lead to changes consistent with EMT, resulting in increased EMT markers such as N-cadherin, α-SMA, and MMP-9, and increased migration and invasiveness ability in vitro and metastasis to distant organs in vivo. This work clearly supports that exosomes can serve as a means of pro-metastatic cross-communicator between different cell populations to achieve cooperative metastasis in ccRCC. In our metastatic models, EMT appears to be an obligatory intermediate state that metastatic cells need to achieve to complete the metastatic cascade. In our preclinical models and clinical tumor tissues, the tumor cells in the lung metastatic lesions exhibit epithelial phenotypes and not mesenchymal, and they are highly proliferative [15]. These results support that the reversal of EMT, namely mesenchymal-to-epithelial transition (MET), is occurring in ccRCC metastasis as consistently observed in the metastatic process of other tumors [40,41,42,43]. Interestingly, EMT induction by the VHL(−) exosomes appeared to be incomplete, as the downregulation of epithelial marker, E-cadherin, and the upregulation of master EMT transcription factors (Snail, Slug, and Zeb) were not observed (Figure 2A). These results suggest that VHL(−) exosomes could be inducing an intermediate EMT state, which could facilitate MET and the reversal to the epithelial state in the final metastatic destination.

This work opens up many interesting clinical investigative issues to pursue. An immediate issue is how to detect the exosomes produced by ccRCC tumor cells and more specifically, the VHL(−) exosomes in patients’ blood. Although tetraspanins CD63 and CD81 are common surface markers of exosomes, they are ubiquitous and unable to selectively identify RCC exosomes. The great challenge to capture RCC-specific exosomes requires the use of cell-specific markers. CD70 is a protein belonging to the tumor necrosis factor superfamily 7 (TNFSF7) that induces cytotoxic effects on B and T lymphocytes leading to immune escape [44]. From the TCGA database, CD70 is most highly expressed in ccRCC amongst all solid tumors. Further, the expression of CD70 of 330 RCC specimens has been studied, and the mean expression was almost doubled in tumor samples compared to normal tissues. Within tumor samples, CD70 expression was higher in sarcomatoid and clear-cell tumors than in oncocytomas and papillary tumors [44,45]. Importantly, CD70 expression is controlled by HIF [46] and is thus upregulated in VHL(−) cells. Thus, it is very promising that we observed the presence of CD70 in both cell lysates and exosomes from VHL(−) cells (Supplementary Figure S1). CD70 could represent a potential specific marker to aid in the capturing and interrogation of RCC exosomes. Furthermore, another ccRCC-specific marker to consider is carbonic anhydrase IX (CAIX), which is one of fourteen carbonic anhydrase enzyme isoforms occurring in humans and has been previously detected in exosomes released from RCC cell lines [47]. CAIX expression is regulated by HIF1α and plays a role in the pH regulation of the cell environment, making the extracellular space more acidic. In ccRCC, due to the loss of VHL and the constitutive activation of HIF1, CAIX is also constitutively expressed, while other renal cell cancers such as chromophobe RCC and papillary RCC are typically CAIX negative [48].

Further investigation of the metastatic crosstalk mediated by RCC exosomes will require the preservation and interrogation of the bioactive molecular cargo in circulating RCC exosomes. To this end, advanced technology such as microfluidic “click beads” could be used to isolate exosomes, followed by reverse transcription digital PCR to identify the nucleic acid bioactive, molecular instructive signals, delivered by exosome payload [49]. 

In addition to diagnostic potential, cancer-specific exosomes and their bioactive contents provide a potential therapeutic target for metastatic disease in response to an initial diagnosis or long-term preventative measures in patients with a high risk of developing RCC [50]. Two possibilities currently being pursued in research are targeting cancer-specific exosomes to impede their communication abilities or using them to deliver therapeutic agents such as chemotherapies for specific and targeted drug release. Targeting cancer-specific exosomes to reduce their metastatic potential aims to eliminate exosomes from circulation, inhibit their secretion, or prevent their internalization into recipient cells [51]. Limitations of exosome inhibition include side effects arising from stopping EV release from healthy cells, and therapies would need development to specifically target tumor cell-derived exosomes. Nonetheless, early and consistent diagnosis of metastatic disease and targeted therapeutic delivery will improve patient outcomes and provide insights into the metastatic stage of other cancers.

## 4. Materials and Methods

### 4.1. Cell Culture

The Renca (RC) murine and the ACHN (AC) human cell lines were purchased from ATCC and engineered to create RC-VHL(+), RC-VHL(−), AC-VHL(+), and AC-VH(−) lines [15]. VHL(+)-*loxP* and VHL(−)-Cre lines were established by lentiviruses packaged from pLV-CMV-LoxP-DsRed-LoxP-eGFP (Addgene, Watertown, MA, USA, #65726) and pcDNA3.1-CFP;Cre25nt;Zeo (Addgene, Watertown, MA, USA, #65727), respectively, in RC-VHL(+) and RC-VHL(−) cells [33,34] (Table 1). All cell lines were cultured in RPMI-1640 medium (Gibco, Waltham, MA, USA, #:11875-093) supplemented with 10% [*v*/*v*] heat-inactivated Fetal Bovine Serum (Genesee Scientific, El Cajon, CA, USA, #25-550), and 1% [*v*/*v*] Penicillin and Streptomycin (Gibco, #15140-122). Exosome-depleted FBS was collected after bovine exosomes were removed via ultracentrifugation at 100,000× *g* for 16 h in an SWI-32-Ti swing bucket rotor (Beckman Coulter, Brea, CA, USA, #L-100XP). 

### 4.2. Exosome Isolation, Imaging, and Quantification

RC-VHL(+), RC-VHL(−), RC-VHL(+)-*loxP*, and RC-VHL(−)-Cre cells in T175 flasks were allowed to reach 60% confluency while AC-VHL(+) and AC-VHL(−) cells were allowed to reach 70% confluency. At this time, the supernatant was discarded, then refreshed with RPMI-1640 medium with exosome-depleted FBS after three sequential PBS (Gibco, #14190-144) washes and incubated over 42 and 72 h, respectively. After the incubation period, the cell supernatant was subjected to sequential centrifugation according to [publication]. Briefly, it was first centrifuged at 300× *g* for 10 min and 2000× *g* for 10 min in a benchtop centrifuge (Eppendorf, Hamburg, Germany, #5804) to remove cells and debris. The supernatant was then ultracentrifuged at 4 °C at 10,000× *g* in an SWI-32-Ti swing bucket rotor (Beckman Coulter) for 30 min to remove proteins. Subsequently, the supernatant was ultracentrifuged at 4 °C at 100,000× *g* for 70 min to pellet exosomes, followed by resuspension in cold PBS and ultracentrifuged at 100,000× *g* for 1 h 10 min at 4 °C to remove the remaining impurities. The exosome pellets were then collected by resuspension in PBS and stored at −80 °C.

The exosome morphology was identified by an FEI T20 transmission electron microscope (TEM) (FEI Company, Hillsboro, OR, USA). Briefly, a 5 μL droplet of exosomes suspension (300 μg/mL) was applied onto a glow-discharged grid coated with carbon film (Ted Pella, #01814-F) and left on the grid for 30 s, followed by negative staining with 2% uranyl acetate. The TEM images of isolated exosomes were identified at 29,000× magnification in a Tecnai TF20 electron microscope (FEI, Hillsboro, OR, USA) operated at 200 kV. Dynamic light scattering (DLS) was used to determine the size and purity of collected exosomes [26]. To determine the number of exosomes per 10 ^6^ cells, cells were counted using a hemacytometer right before exosome isolation, and exosome concentration was determined using the bicinchoninic acid (BCA) Rapid Gold Protein Assay kit (Thermo Scientific, Waltham, MA, USA, #A53226)

### 4.3. Internalization of PKH67-Labeled VHL-Knockout Exosomes

Visualization of exosome internalization was completed using freshly isolated exosomes. PKH67 (Millipore Sigma, Burlington, MA, USA, #MINI67) was added to 100 μg exosomes and a control containing Diluent C; then, the volume was supplemented with PBS. Stained exosomes were then isolated via ultracentrifugation at 4 °C 100,000× *g* for 70 min. Exosome pellets were resuspended in RPMI-1640 medium containing exo-free FBS. Then, 2.50 μg exosomes were added to each well of a 48-well plate with fresh RPMI-1640 exosome-free medium, and cells were incubated at 37 °C and 5% CO_2_ for 24 h. After fixed with 10% formalin (Epredia, Kalamazoo, MI, USA, #5701), cell nuclei were stained with 4′,6-diamidino-2-phenylindole (DAPI; Invitrogen, Waltham, MA, USA, #D1306), and F-actin was monitored by rhodamine-phalloidin (Invitrogen, #R37112) staining. Images were taken with the NIS-Elements AR imaging program on a fluorescent microscope (Nikon ECLIPSE Ti, Tokyo, Japan) at 20× magnification and a confocal microscope (Carl Zeiss, LSM880, Oberkochen, Germany) at 10× magnification.

### 4.4. Cre-loxP Color Conversion Assay

Baseline RFP fluorescence of VHL(+)-*loxP* cells, as well as their conversion to GFP upon uptake of VHL(−)-Cre exosomes, was quantified by flow cytometry using BD LSRII SORP Flow Cytometer (BD Biosciences, San Jose, CA, USA). For imaging, 1.0 × 10^5^ VHL(+)-*loxP* cells were seeded onto 12-well plates. After cell adhesion overnight, the medium was discarded and replaced with RPMI-1640 medium containing exosome-depleted FBS containing VHL(−)-Cre exosomes in experimental groups and RPMI-1640 medium with exosome-depleted FBS in control groups. Images were taken at 20× magnification using the NIS-Elements AR imaging program on a fluorescent microscope (Nikon). For flow cytometry, cells were treated as described in Supplementary Materials and Methods Section 3, then trypsinized after 48 h for analysis.

To determine the color conversion state of migratory cells, transwell assays were conducted with 3.0 × 10^4^ cells VHL(+)-*loxP* on the top chamber with no-FBS RPMI-1640 medium, and the bottom chamber filled with RPMI-1640 containing exosome-depleted FBS. After adding 10 μg of VHL(−)-Cre exosomes (experimental group) or RPMI-1640 medium (no exo group), VHL(+)-*loxP* cells were incubated for 48 h. After being fixed with methanol (Fisher Scientific, Waltham, MA, USA, #A412-4) and removal of non-migratory cells as described in Supplementary Materials and Methods Section 3, migrated cells on the bottom side of the transwell chambers were imaged at 100× with a light and fluorescent microscope (Nikon), then analyzed by flow cytometry using BD LSRII SORP Flow Cytometer (BD Biosciences) for RFP and GFP as described above.

### 4.5. Statistical Analysis

Statistical analysis was performed using the Prism software (GraphPad, San Diego, CA, USA, version 9.3.1). Quantitative data are displayed using means ± standard deviations (SD). Statistical significance was determined by a Student’s *t*-test when there were two groups or by one-way ANOVA when there were three or more groups. A *p*-value cutoff of 0.05 was used to establish significance and shown using asterisks (* *p* < 0.05, ** *p* < 0.01, *** *p* < 0.001, **** *p* < 0.0001).

## 5. Conclusions

Intratumoral crosstalk between cancer cells with pVHL and those without is essential for distant metastasis in RCC. Exosomes produced by VHL(−) RCC cells can induce EMT, migration, invasion, and distant metastasis in VHL(+) RCC cells upon uptake. Thus, exosomes play significant roles in mediating malignant cooperative communication and can be an important diagnostic and therapeutic target.

## Figures and Tables

**Table 1:** The cell line information: parental cells and modifications made.

Cell Line	Parental Cells	Modifications
RC-VHL(+)	Renca	VHL wildtype, RFP; as described in [15]
RC-VHL(−)	Renca	VHL knockout, GRP; as described in [15]
AC-VHL(+)	ACHN	VHL wildtype; as described in [15]
AC-VHL(−)	ACHN	VHL knockout; as described in [15]
RC-VHL(+)-*loxP*	RC-VHL(+)	RFP → GFP upon receiving Cre; lentivirally overexpressed plasmid from Addgene#65726 [33,34]
RC-VHL(−)-Cre	RC-VHL(−)	Lentivirally overexpressed plasmid from Addgene#65727 [33,34]

## Data Availability

The data used or analyzed during the current study are available from the corresponding author. The AssayAnalyze program is available on GitHub (https://github.com/ebowen19/AssayAnalyze, accessed on 17 June 2023).

## Supplementary Materials

The following supporting information can be downloaded at: https://www.mdpi.com/article/10.3390/ijms242417307/s1.

## Abbreviations

RCC	Renal cell carcinoma
ccRCC	clear cell renal cell carcinoma
CAM	Chorioallantoic membrane model
DLS	Dynamic light scattering
DAPI	4′,6-diamidino-2-phenylindole
GAPDH	Glyceraldehyde-3-phosphate dehydrogenase
BCA	Bicinchoninic acid
qPCR	quantitative PCR
RT-qPCR	Reverse transcription qPCR
SD	Standard deviation
MMP-9	Matrix metalloproteinase 9
EMT	Epithelial–mesenchymal transition
RFP	Red fluorescent protein
GFP	Green fluorescent protein
