# Supplementary material for: Exosomes from Von Hippel-Lindau-Null Cancer Cells Promote Metastasis in Renal Cell Carcinoma
Source: Int J Mol Sci. 2023 Dec 9;24(24):17307. doi: 10.3390/ijms242417307 (PMC10743428; doi:10.3390/ijms242417307)
Supplement: Supplementary file 1 [file ijms-24-17307-s001.zip › Supplementary figures.pdf]

Supplementary Figures

Supplementary Figure S1

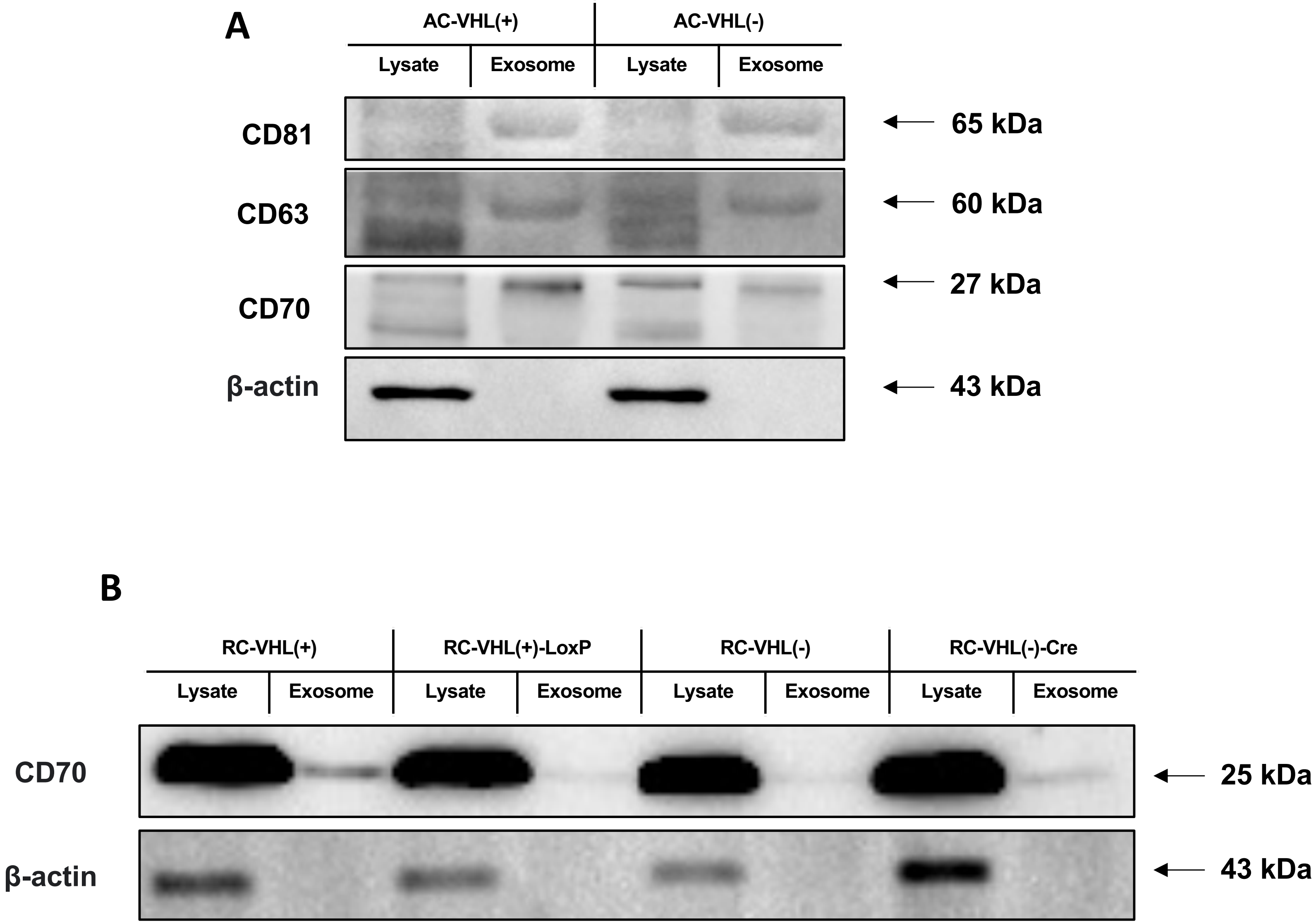

c

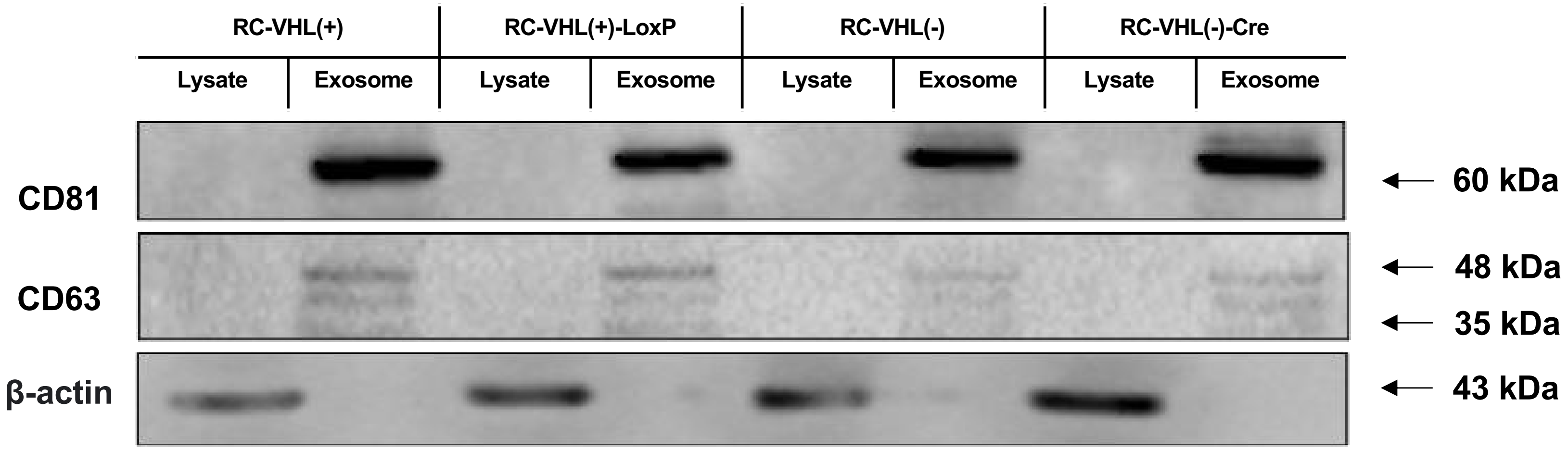

Supplementary Figure S1. Western blots of tumor-derived exosomes. a AC-VHL(+) and AC-VHL(-) cell lysates and exosomes all express CD70, a marker of ccRCC, while exosomes exclusively express exosome-specific markers, CD81, and CD63. β-actin is expressed in only the cell lysates. **b** and **c** show similar expression patterns for RC-VHL(+), RC-VHL(+)-LoxP, RC-VHL(-), and RC-VHL(-)-Cre lysates and exosomes.

Supplementary Figure S2

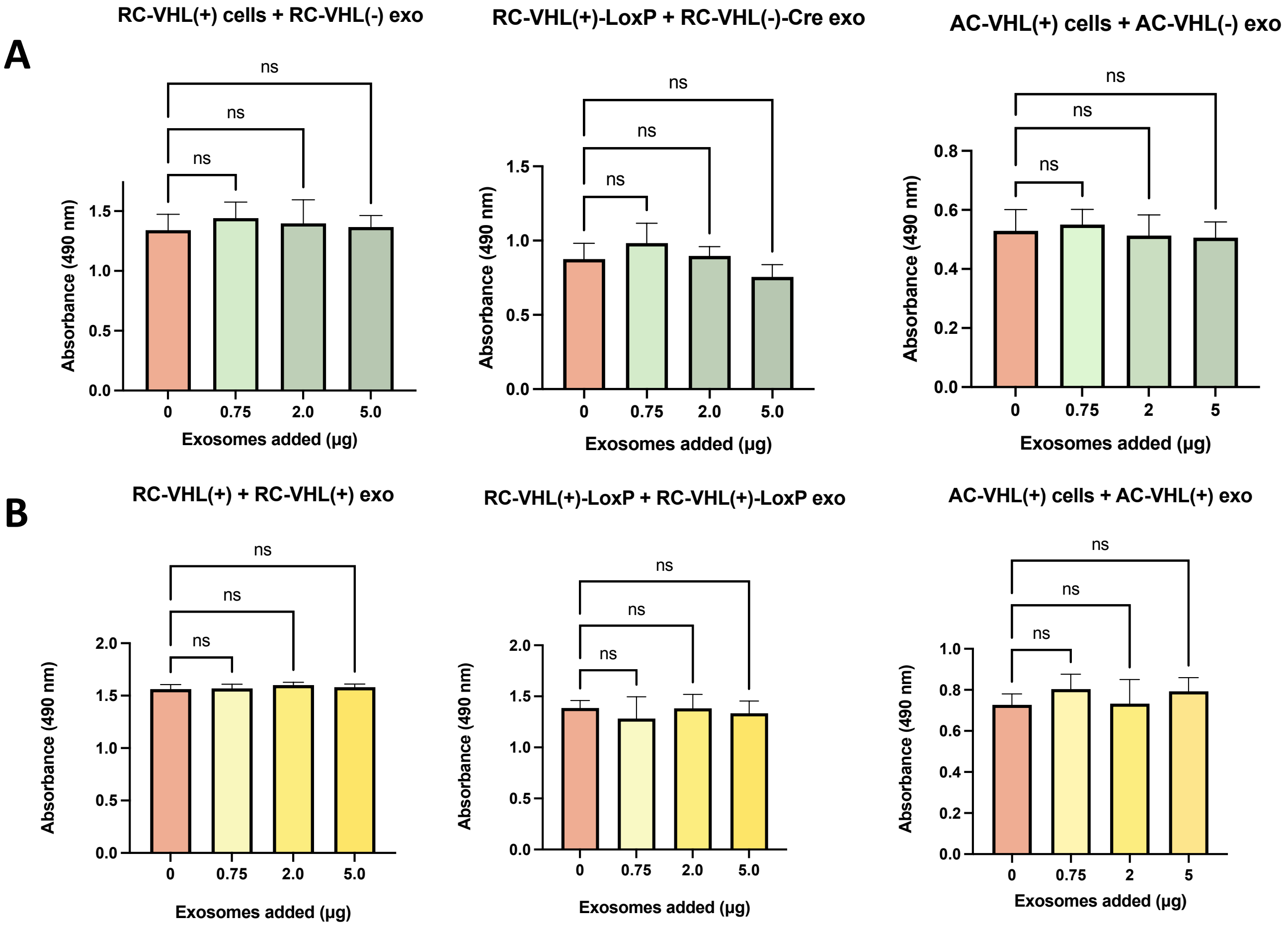

Supplementary Figure S 2. Tumor-derived exosomes have no significant effects on cell proliferation. Cell Proliferation of VHL(+) cells; RC-VHL(+), RC-VHL(+)-LoxP, and AC-VHL(+) cells, in response to **a** VHL(-) and **b** VHL(+) exosomes measured by absorbance at 490 nm. Statistical analysis completed using Ordinary one-way ANOVA. (*p < 0.05)

Supplementary Figure S3

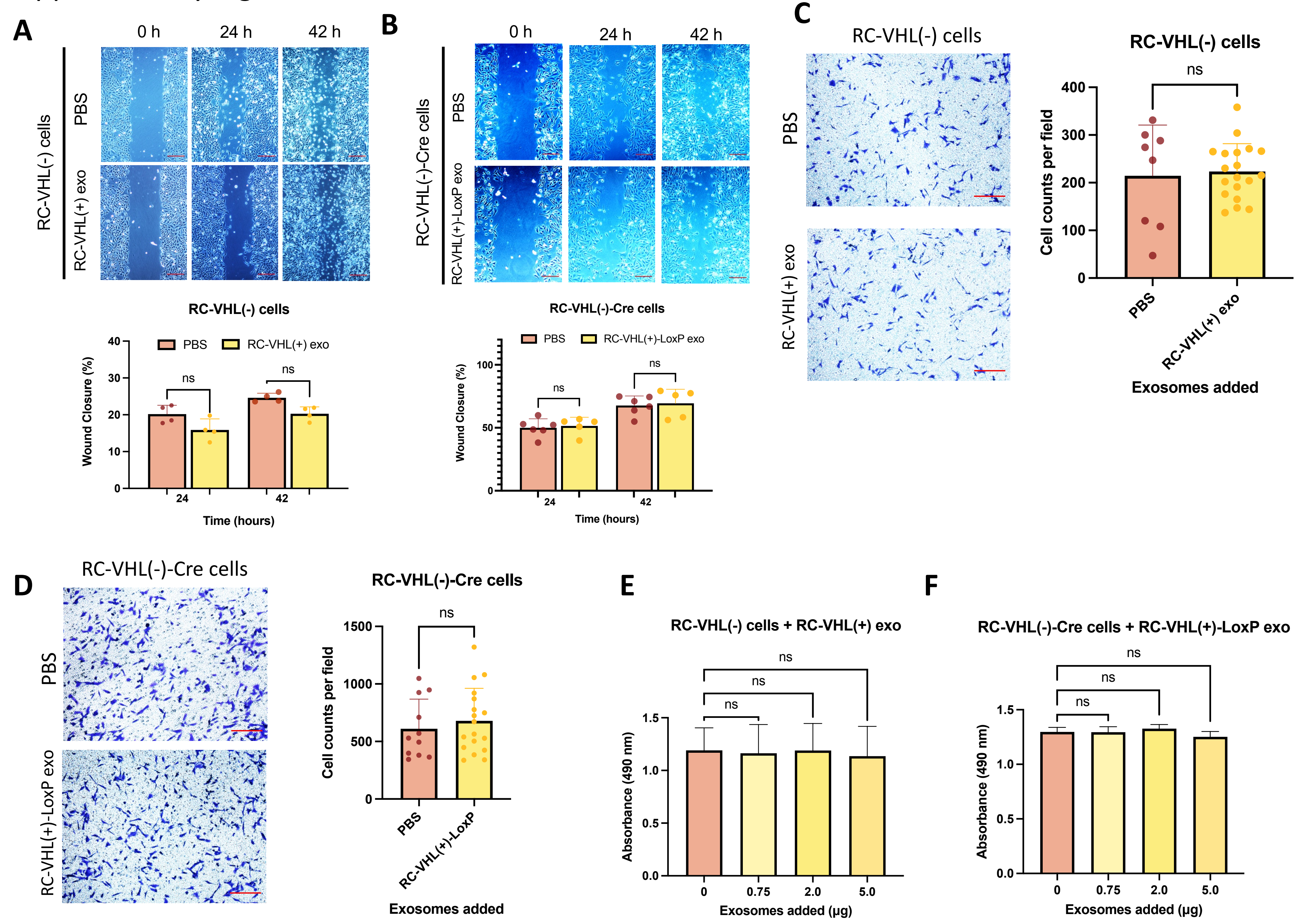

Supplementary Figure S 3. Behavior of VHL(-) cells is unchanged when treated with VHL(+) exosomes. **a** Wound Healing assay of RC-VHL(-) cells in response to RC-VHL(+) exosomes and **b** RC-VHL(-)-Cre cells in response to RC-VHL(+)-LoxP exosomes over the course of 42-hour incubation. No significant changes in wound closure and two-dimensional migration were observed. Transwell migration assays of **c** RC-VHL(-) cells in response to RC-VHL(+) exosomes and **d** RC-VHL(-)-Cre cells in response to RC-VHL(+)-LoxP exosomes after 36-hour incubation. No significant changes in chemotactic migration were observed. **e** Proliferation of RC-VHL(-) cells in response to RC-VHL(+) exosomes and **f** RC-VHL(-)-Cre cells in response to RC-VHL(+)-LoxP exosomes measured by change in absorbance at 490 nm. VHL(+) exosomes had no significant effect on proliferation of VHL(+) cells. Imaged at 10x magnification on a light microscope. Scale bars: (**a**)(**b**)(**c**)(**d**) 500 μm. Statistical analysis completed using (**a**) (**b**) (**e**) (**f**) Ordinary one-way ANOVA and (**c**) (**d**) Welch's t-test (*$p < 0.05$).

Supplementary Figure S4

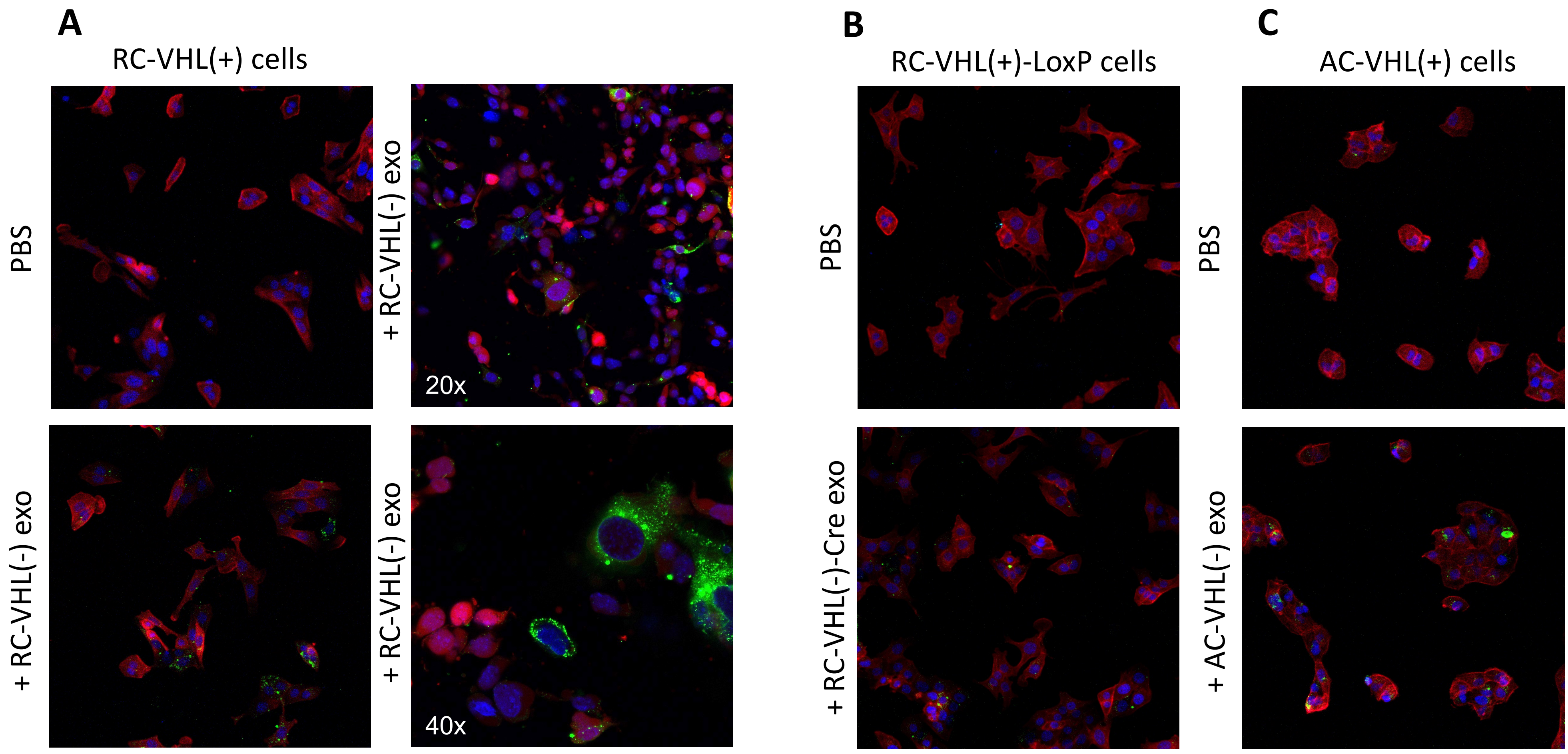

Confocal images (20x top, 40x bottom)
RC-VHL

Supplementary Figure S4. Confocal microscopy shows VHL(-) exosomes are internalized into VHL(+) cells. Three-dimensional confocal imaging of **a** RC-VHL(+) cells with PBS or RC-VHL(-) exosomes at 10x, 20x, and 40x. **b** RC-VHL(+)-LoxP cells with PBS or RC-VHL(-)-Cre exosomes and **c** AC-VHL(+) cells with PBS or AC-VHL(-) exosomes. Images were taken unless otherwise specified at 10x magnification.

Supplementary Figure S5

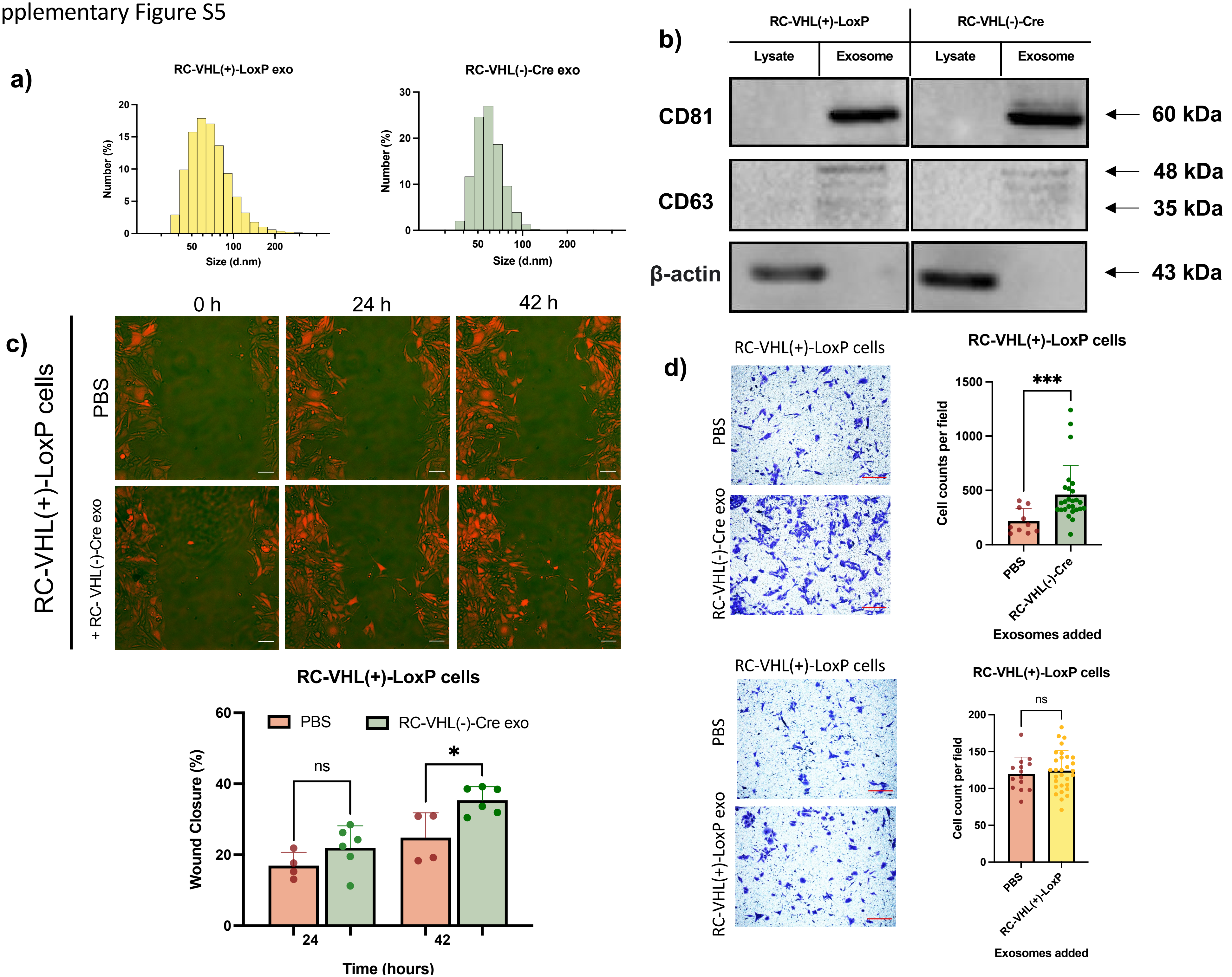

Supplementary Figure S5. The Cre-LoxP variants, RC-VHL(+)-LoxP and RC-VHL(-)-Cre, of the RENCA cell line show the same characteristics and experimental outcomes as the standard RC-VHL(+) and RC-VHL(-) cells. **a** Dynamic Light Scattering of tumor-derived exosomes; RC-VHL(+)-LoxP and RC-VHL(-)-Cre exosomes. **b** RC-VHL(+)-LoxP and RC-VHL(-)-Cre exosomes exclusively express exosome-specific markers, CD81, and CD63. β-actin is expressed in only the cell lysates. **c** RC-VHL(+)-LoxP cells in response to PBS or RC-VHL(-)-Cre over the course of 42-hour incubation show significantly more two-dimensional migration in the RC-VHL(-)-Cre-treated groups than in PBS groups. Imaged at 20x on a fluorescent microscope. **d** Transwell migration assays of RC-VHL(+) cells in response to RC-VHL(-)-Cre exosomes and RC-VHL(+) exosomes after 36-hour incubation. Increased migratory capacity was observed in the RC-VHL(-)-Cre-treated group. Scale bars: (**c**) 100 µm (**d**) 500 µm. Statistical analysis completed using (**c**) Ordinary one-way ANOVA and (**d**) Welch's t-test (*$p < 0.05$, ***$p < 0.001$).

Supplementary Figure S6

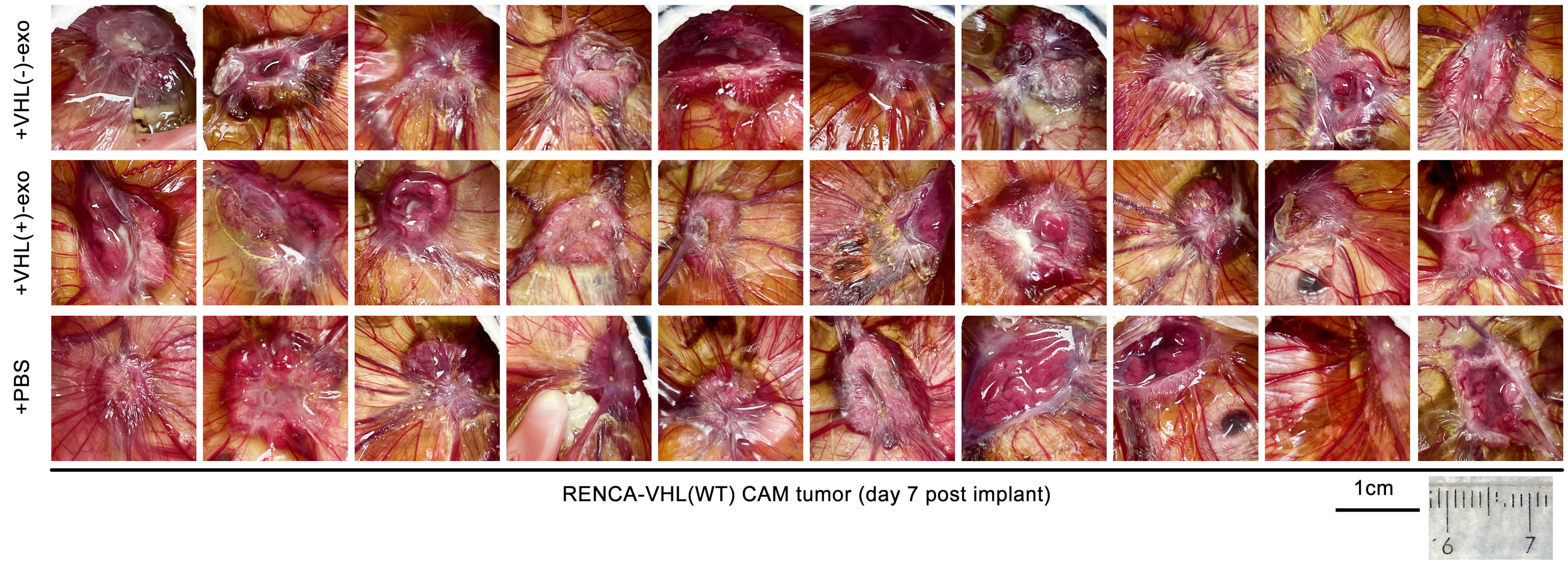

Supplementary Figure S6. Gross view of RC-VHL(+) tumors formed in dCAM before dissection. No visible differences were detected.
