# Supplementary material for: Exosomes from Von Hippel-Lindau-Null Cancer Cells Promote Metastasis in Renal Cell Carcinoma
Source: Int J Mol Sci. 2023 Dec 9;24(24):17307. doi: 10.3390/ijms242417307 (PMC10743428; doi:10.3390/ijms242417307)
Supplement: Supplementary file 1 [file ijms-24-17307-s001.zip › Supplementary Methods final.pdf]

Supplementary materials and methods

1. Western Blotting

Samples containing cells or exosomes were lysed with cOmplete™, Mini Protease Inhibitor Cocktail (Millipore Sigma, Burlington, MA, USA, #11836153001). Protein concentration was determined with the BCA protein kit. SDS/PAGE gels were prepared using 30% Acrylamide/Bis Solution with a 37:5:1 crosslinker ratio (Bio-Rad, Hercules, CA, USA, 1610158), and 20 μg cell lysates and exosomes were added to each well and ran against Tri-color Protein Marker I (MP Biomedicals, Irvine, CA, USA, #PM01) and Page Ruler Prestained Protein Ladder (Thermo Scientific, #26616) for size determination. After band separation, the gels were transferred to nitrocellulose membranes (Bio-Rad, #1620112). Membranes were then subjected to a TBST blocking buffer containing 5% (w/v) Bovine Serum Albumin (BSA) (Bio-Rad, #170-6404) and incubated in primary antibodies (β-actin, CD81, CD63, and CD70; see below) at 4°C overnight. Following incubation, membranes were washed with TBST three times before the secondary antibody (mouse, rabbit) (Jackson ImmunoResearch, West Grove, PA, USA) was incubated for 1 h at room temperature. Bands were visualized by enhanced chemiluminescence with a Bio-Rad ChemiDoc XRS+ imaging system. The primary antibodies used are as follows: β-actin (Santa Cruz Biotechnology, Dallas, TX, USA, #sc-47778, 1:2000), CD81 (Novus Biologicals, Centennial, CO, USA, #NB100-65805, 1:1000), CD63 (Novus Biologicals, #NB100-77913, 1:1000) and CD70 (Invitrogen, Waltham, MA, USA, #PA5-102557).

2. Wound Healing Assay

12-well plates were seeded with 2.5 x 10^5 cells and allowed to reach 90% confluence. A p-10 pipette tip was used to scratch a straight line of cells out of the center of the plate. The medium was then replaced with RPMI-1640 medium lacking FBS with VHL wildtype or VHL knockout exosomes added in. Pictures were taken over 42 h at 10x magnification. To perform the computational wound healing analysis, AssayAnalyze (https://github.com/ebowen19/AssayAnalyze), a program developed by the Wu Lab, was used. The program identifies the gap region in an image and quantifies the number of pixels it contains. First, the images were thresholded to create a binary image with distinct background and foreground regions using either adaptive or variance-based methods. Next, any loose cells in the gap region were removed through erosion and dilation operations. These images were subtracted to obtain gap boundaries, which were filled and refined to remove remaining noise. Next, the gap was segmented into one distinct foreground object through a series of dilation and erosion, area opening, and hole-filling operations with the smallest structuring element that would create a single foreground object for each image. Next, the program calculated the number of pixels in the single foreground object to quantify the gap size. The program was applied to the images at hours 0, 24, and 42 h to determine the percent migration in each sample.

Fluorescence microscopy was completed via time-lapse to measure color conversion and migratory effects arising from exosome internalization over a 45h period via time-lapse imaging at 20x magnification. Data analysis was completed using Adobe Photoshop (San Jose, CA, USA, ver. 23.5.2) to measure the free area between the cell borders at hours 0 and 45 h to determine the percent migration in each sample by pixel count.

3. Transwell Assays

6.5 mm diameter, 8.0 um pore size, and 24-well membrane inserts from Corning (Corning, NY, USA, #3422) were used for transwell migration assays. 3.0 x 104 cells were seeded into the upper chambers in RPMI-1640 medium lacking FBS. Lower chambers contained RPMI-1640 medium with exosome-depleted FBS and exosomes derived from VHL-wildtype or VHL-knockout cells. After 36 or 50 h of incubation, for Renca and ACHN cell lines, respectively, the transwell inserts were fixed with methanol (Fisher Scientific, Waltham, MA, USA, #A412-4) and stained with 1% crystal violet (Fisher Scientific, #C581-25). Nonmigratory cells were removed using cotton swabs. Cell counts were done in random fields under a light microscope (Nikon ECLIPSE Ti, Tokyo, Japan) at 10x magnification. Invasion assays were completed using 50 μL of RPMI-1640 medium and Matrigel (Corning, #354234) at a ratio of 10:1 that was allowed to solidify for 1 h before cell seeding.

For the computational analysis of the transwell assay images, the cell counting script from AssayAnalyze was used. The program calculated local variance at different window sizes to identify blurry regions, which were stored in a binary mask. A brightness threshold is determined to separate the foreground (cells) from the background. The program combined the blurry regions with the foreground to obtain a binary image with clear cell foreground regions. To remove noise, small specks were removed using an area opening. Cell counting was performed by applying erosion to separate any connected cells and labeling connected components. The program then counted the number of distinct foreground objects representing individual cells.

4. RNA Isolation and qPCR for EMT Markers

RNA was extracted from 6-well plates using TRIzol (Invitrogen, #15596018) and chloroform (Fisher Scientific, #C298-500) for liquid-liquid extraction after a 50h incubation with exosomes. After RNA was isolated, cDNA was generated by reverse transcription using Takara PrimeScript RT Reagent Kit (Perfect Real Time) (Kusatsu, Shiga, Japan, #RR037A) on a thermal cycler (MJ Research, Deltona, FL, USA, #PTC-200) according to manufacturer'ser's protocol. Then, SYBR qPCR (Meridian Bioscience, Cincinnati, OH, USA, # QT615-05) was employed to compare the RNA expression of EMT markers in each sample on the QuantStudio 5 instrument (Applied Biosystems, Foster City, CA, USA, #A34322). Specific primers (see below table) were purchased from Integrated DNA Technologies (Coralville, IA, USA). The relative expression levels of mRNA were normalized to glyceraldehyde-3-phosphate dehydrogenase (GAPDH) using the 2−ΔΔCt method.

Gene	Primer	Sequence
GAPDH	F	AGGTCGGTGTGAACGGATTTG
	R	TGTAGACCATGTAGTTGAGGTCA
E-cadherin	F	CAGGTCTCCTCATGGCTTTGC
	R	CTTCCGAAAAGAAGGCTGTCC
N-cadherin	F	AGCGCAGTCTTACCGAAGG
	R	TCGCTGCTTTCATACTGAACTTT
α-SMA	F	GTCCCAGACATCAGGGAGTAA
	R	TCGGATACTTCAGCGTCAGGA
SNAI1	F	CACACGCTGCCTTGTGTCT
	R	GGTCAGCAAAAGCACGGTT
SNAI2	F	CAGCGAACTGGACACACACA
	R	ATAGGGCTGTATGCTCCCGAG

Zeb1	F	ACCGCCGTCATTTATCCTGAG
	R	CATCTGGTGTTCCGTTTTCATCA
Zeb2	F	AAACGTGGTGAACTATGACAACG
	R	CTTGCAGAATCTCGCCACTG
MMP-9	F	CTGGACAGCCAGACACTAAAG
	R	CTCGCGGCAAGTCTTCAGAG

* F: Forward primer (5'-3'); R: Reverse primer (3'-5')

5. Cell Proliferation Assay

1.0 x 105 cells were seeded into each well of a flat bottom 96-well plate and incubated for 40 h for Renca cell lines and 48 h for ACHN cell lines at 37°C and 5% CO2. Exosomes were added in a concentration array of 0, 0.75, 2.0, and 5.0 µg and returned to the incubator. After 42 or 48 h, the medium was discarded and replaced with MTS (Promega, Madison, WI, USA, #G3580) diluted 1:10 in RPMI-1640 (no FBS) medium. After 2h incubation, absorbance was measured at a wavelength of 490 nm using a CLARIOstar Plus plate reader (BMG Labtech, Ortenberg, Germany).

6. Duck Chorioallantoic Membrane (dCAM) Model

Fertilized duck eggs were purchased from AA Laboratory Eggs (Westminster, CA, USA) and processed as previously described [1–4]. Briefly, windows were opened on developmental day 14 and sealed with Tegaderm (3M, Saint Paul, MN, USA, #1624W & #1634). On developmental day 14 (tumor day 0), 3.0 x 106 RC-VHL(+) cell lines suspended in 20 µL RPMI-1640 medium with 10% FBS and 1% Penicillin and Streptomycin were implanted onto a vascular-rich area of the chorioallantoic membrane by using a coarse glass rod to disrupt the ectoderm and make the surface rough for implantation. Three experimental groups were established: RC-VHL(+) cells treated with 1) PBS, 2) RC-VHL(+)-derived exosomes, and 3) RC-VHL(-)-derived exosomes. PBS or exosomes were added on developmental days 17, 19, and 21 (tumor days 3, 5, and 7) in 80 µg doses directly to the tumor surface. These number of exosomes and frequent treatment (every 48 h) were to account for high cell numbers in the tumor, possible minimal effective internalization, and the possible loss of exosome suspension to non-tumor areas. On developmental day 28 (tumor day 14), eggs were euthanized after being anesthetized by putting them on ice for 10min. Gross pictures of dCAM tumors were taken on developmental day 28 either in ovo before euthanasia or dissected out after euthanasia. Tumor weights and sizes were also measured at this time. Embryonic livers were collected for analysis via qPCR as previously described [1–4]. Briefly, genomic DNA was extracted from the liver samples by phenol-chloroform (Fisher Scientific, #BP1752I-400) liquid-liquid phase extraction, precipitated with 100% ethanol and 70% ethanol to remove salts. After 5 min centrifugation at 12,000 x g to pellet the genomic DNA, samples were resuspended in ddH2O. qPCR was done on the extracted genomic DNA using TaqMan™ Fast Advanced Master Mix (Applied Biosystems, #4444557) and TaqMan™ probes for avian and mouse β-actin (Applied Biosystems, #433182, Assay ID: Gg03815934_s1 ACTB and Mm02619580_g1 Actb, respectively). The reaction mixture was amplified on the QuantStudio 5 instrument (Applied Biosystems). Relative fold expression of mouse β-actin to avian β-actin was determined using the 2-ΔΔCt method.

References

1. Zhang, Z.; Hu, J.; Ishihara, M.; Sharrow, A.C.; Flora, K.; He, Y.; Wu, L. The MiRNA-21-5p Payload in Exosomes from M2 Macrophages Drives Tumor Cell Aggression via PTEN/Akt Signaling in Renal Cell Carcinoma. *Int. J. Mol. Sci.* **2022**, *23*, 3005, doi:10.3390/ijms23063005.

2. Hu, J.; Ishihara, M.; Chin, A.I.; Wu, L. Establishment of Xenografts of Urological Cancers on Chicken Chorioallantoic Membrane (CAM) to Study Metastasis. *Precis. Clin. Med.* **2019**, *2*, 140–151, doi:10.1093/pcmedi/pbz018.

3. Sharrow, A.C.; Ishihara, M.; Hu, J.; Kim, I.H.; Wu, L. Using the Chicken Chorioallantoic Membrane In Vivo Model to Study Gynecological and Urological Cancers. *J. Vis. Exp.* **2020**, doi:10.3791/60651.

4. Ishihara, M.; Hu, J.; Zhang, X.; Choi, Y.; Wong, A.; Cano-Ruiz, C.; Zhao, R.; Tan, P.; Tso, J.L.; Wu, L. Comparing Metastatic Clear Cell Renal Cell Carcinoma Model Established in Mouse Kidney and on Chicken Chorioallantoic Membrane. *J. Vis. Exp.* **2020**, doi:10.3791/60314.
