# Supplementary material for: Exosomes from Von Hippel-Lindau-Null Cancer Cells Promote Metastasis in Renal Cell Carcinoma
Source: Int J Mol Sci. 2023 Dec 9;24(24):17307. doi: 10.3390/ijms242417307 (PMC10743428; doi:10.3390/ijms242417307)
Supplement: Supplementary file 1 [file ijms-24-17307-s001.zip › supplementary videos/supple video legends.pptx]

## Slide 1
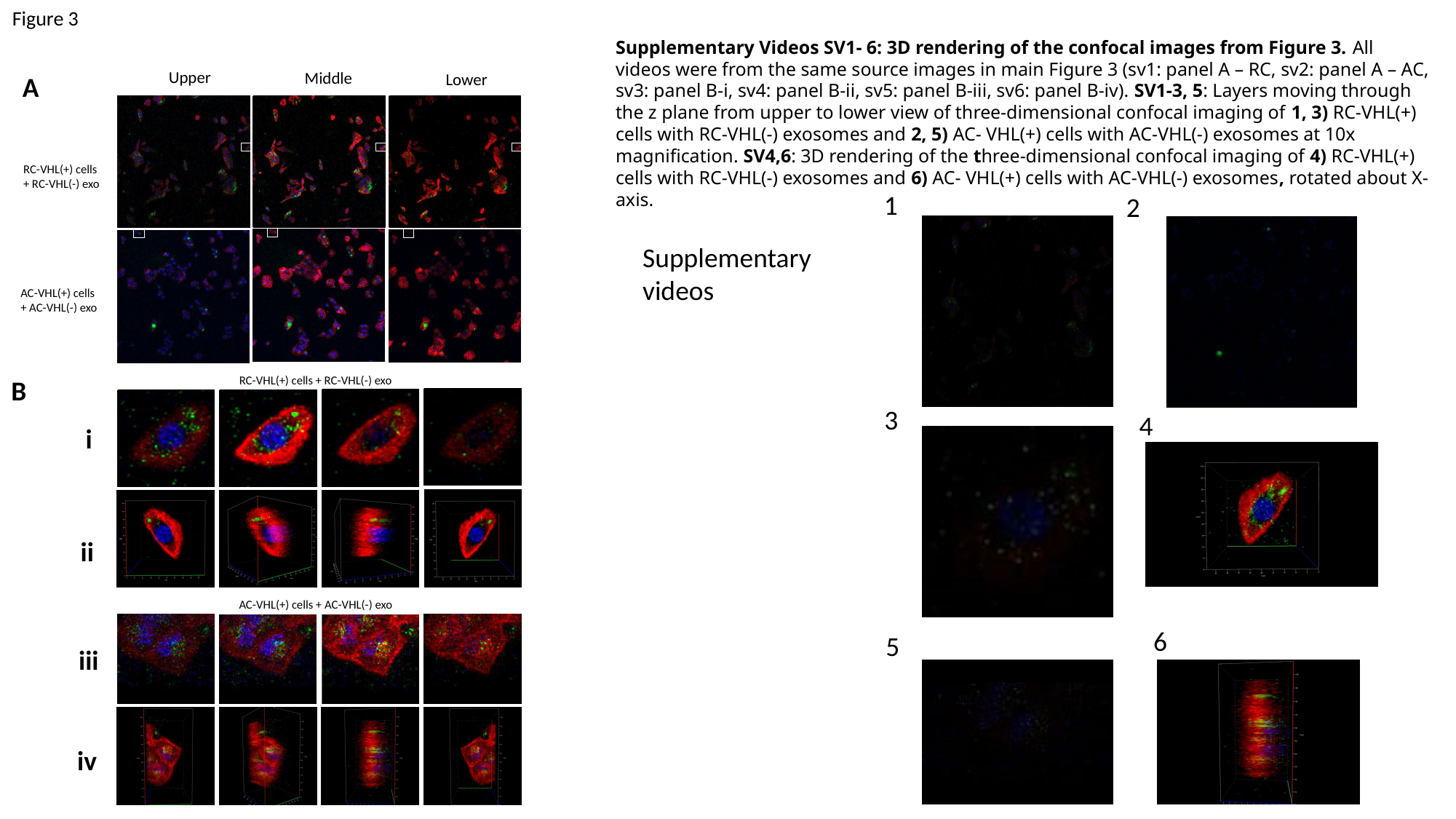

Figure 3
Supplementary Videos SV1- 6: 3D rendering of the confocal images from Figure 3. All videos were from the same source images in main Figure 3 (sv1: panel A – RC, sv2: panel A – AC, sv3: panel B-i, sv4: panel B-ii, sv5: panel B-iii, sv6: panel B-iv). SV1-3, 5: Layers moving through the z plane from upper to lower view of three-dimensional confocal imaging of 1, 3) RC-VHL(+) cells with RC-VHL(-) exosomes and 2, 5) AC- VHL(+) cells with AC-VHL(-) exosomes at 10x magnification. SV4,6: 3D rendering of the three-dimensional confocal imaging of 4) RC-VHL(+) cells with RC-VHL(-) exosomes and 6) AC- VHL(+) cells with AC-VHL(-) exosomes, rotated about X- axis.
Upper
Middle
Lower
A
Upper
Middle
Lower
RC-VHL(+) cells + RC-VHL(-) exo
1
2
Supplementary
videos
AC-VHL(+) cells + AC-VHL(-) exo
RC-VHL(+) cells + RC-VHL(-) exo
B
3
4
i
ii
AC-VHL(+) cells + AC-VHL(-) exo
6
5
iii
iv
